# Prevalence and Determinants of Workplace Violence Against Nurses in the Italian Home Care Settings: A Cross‐Sectional Multicentre Study

**DOI:** 10.1111/jocn.70007

**Published:** 2025-07-01

**Authors:** Manuele Cesare, Marco Di Nitto, Paolo Iovino, Valeria Caponnetto, Yari Longobucco, Ilaria Marcomini, Francesco Zaghini, Rosaria Alvaro, Alessandra Burgio, Giancarlo Cicolini, Loreto Lancia, Paolo Landa, Duilio Fiorenzo Manara, Beatrice Mazzoleni, Laura Rasero, Gennaro Rocco, Maurizio Zega, Loredana Sasso, Annamaria Bagnasco

**Affiliations:** ^1^ A. Gemelli IRCCS University Hospital Foundation Rome Italy; ^2^ Section of Hygiene, Department of Health Science and Public Health Catholic University of the Sacred Heart Rome Italy; ^3^ Centre of Excellence for Nursing Scholarship Board of Nursing (OPI) of Rome Rome Italy; ^4^ Department of Health Sciences University of Genoa Genoa Italy; ^5^ Department of Health Sciences University of Florence Florence Italy; ^6^ Department of Life, Health and Environmental Sciences University of L'Aquila L'Aquila Italy; ^7^ Vita‐Salute San Raffaele University Milan Italy; ^8^ Department of Biomedicine and Prevention University of Rome Tor Vergata Rome Italy; ^9^ Scientific Committee CERSI‐FNOPI Rome Italy; ^10^ Istituto Nazionale di Statistica – ISTAT Rome Italy; ^11^ Department of Innovative Technologies in Medicine & Dentistry “G. d'Annunzio” University of Chieti‐Pescara Chieti Italy; ^12^ Département des Opérations et Systèmes de Décision, Faculté des Sciences de l'Administration Université Laval Québec Canada; ^13^ Axe Santé des Populations et Pratiques Optimales en Santé Centre de Recherche du Centre Hospitalier Universitaire de Québec Québec Canada; ^14^ Groupe de Recherche en Écologie Buccale Université Laval Québec Canada; ^15^ Centre Interuniversitaire de Recherche sur les Réseaux d'Entreprise, la Logistique et le Transport (CIRRELT) Québec Canada; ^16^ Department of Biomedical Sciences Humanitas University Milan Italy; ^17^ FNOPI Board Member Rome Italy; ^18^ Degree Course in Nursing Catholic University “Our Lady of Good Counsel” Tirana Albania; ^19^ Ospedale Isola Tiberina – Gemelli Isola Rome Italy; ^20^ Scientific Director, CERSI‐FNOPI Rome Italy

**Keywords:** epidemiology, home care services, nurses, prevalence, protective factors, risk factors, workplace violence

## Abstract

**Aims:**

To describe the prevalence and determinants of workplace violence against nurses in the Italian home care setting.

**Design:**

Secondary cross‐sectional analysis of data from the multicentre study AIDOMUS‐IT.

**Methods:**

Nurses employed in home care services provided by Italian Local Health Authorities were interviewed using a variety of instruments. A multivariable binary logistic regression model was performed to model the risk of workplace violence against nurses in the last 12 months. Variables related to violence were selected among sociodemographic characteristics (such as age and gender), work‐related factors (including years of experience, team composition, overtime working, previous experience in mental health care, burnout) and organisational elements (including leadership and support, workload, staffing and resources adequacy, and time to reach the patients' homes). Adjusted odds ratios (aOR) were used to present the results.

**Results:**

A total of 3949 nurses participated in the study and 20.49% of them reported to have experienced an episode of violence in the last 12 months. Determinants of higher risk of violence episodes were younger age (aOR = 1.02, *p* = 0.002), higher workload (aOR = 1.01, *p* = 0.002), working in a multiprofessional team (aOR = 1.24, *p* = 0.018), perception of inadequate managerial leadership and support (aOR = 1.38, *p* = 0.003), and higher burnout levels (aOR = 1.01, *p* < 0.001).

**Conclusion:**

The prevalence of workplace violence against Italian home care nurses is high. Several modifiable determinants were found to be associated with a higher risk of violence, which can potentially be mitigated with tailored interventions.

**Implications for the Profession and/or Patient Care:**

Effective preventive strategies must be developed to lessen workplace violence against nurses in the home care setting. These strategies should focus on strengthening nursing managers' leadership and support skills, enhancing team‐building strategies, avoiding inadequate workload, monitoring nurses' burnout, estimating optimum staffing levels, and assigning advanced‐career nurses to home care services. These measures are imperative to guarantee the quality and safety of home care organisations and to attain favourable outcomes in the provision of care.

**Impact:**

This study aimed to explore the prevalence and determinants of workplace violence against nurses in the Italian home care settings. We found that out of the 3949 nurses surveyed, 20% of the sample reported one episode of violence during the last 12 months. Determinants of this violence included younger age, higher workload and burnout, being in a multiprofessional team, and perception of lack of leadership and support by the nurse manager. The results of this study can be used to tailor interventions aimed at mitigating the risk factors of violence, particularly those that can be modified (e.g., workload, burnout, and leadership).

**Reporting Method:**

The study adhered to the Strengthening the Reporting of Observational Studies in Epidemiology (STROBE) guidelines.

**Patient or Public Contribution:**

No patient or public contribution.

**Summary**

- What does this paper contribute to the wider global clinical community?
  ○ Workplace violence against nurses in home care settings is common and associated with younger age, as well as several modifiable work‐related variables including high workload, burnout, working with other non‐nurse health care providers, and perception of inadequate nurse managers' leadership.
  ○ The modifiable risk factors identified in this study can be mitigated through targeted interventions designed to reinforce managerial leadership, decrease workload and effectively prevent and manage burnout among nursing teams working in home care settings.

## 1 Introduction

Risky conditions hinder the provision of high‐quality patient care. Although violence can occur in a variety of sectors and professions, the healthcare setting is disproportionately affected by this problem, with nurses being frequently involved in such events (Pagnucci et al. 2022). Contrary to common belief, supporting vulnerable individuals and providing care to others exposes nurses to significant dangers (Escribano et al. 2019). Aggression, harassment and workplace violence (WPV) against nurses who provide direct patient care are commonplace and directly associated to unfavourable outcomes for nurses, patients, and organisations (Kafle et al. 2022).

The recent outbreak of COVID‐19 has led to a concerning rise in WPV, which poses a serious threat to the sustainability of the healthcare system and the emotional well‐being of nurses (Molero Jurado et al. 2023). Consequently, health care settings have turned into hostile work environments, where nurses are regularly ‘assaulted and unheard’, transforming them from ‘heroes’ into ‘wounded heroes’ who do not get any credit or official support out of the caring process (Escribano et al. 2019). Furthermore, due to the aging population, the coexistence of multiple diseases, and the surge in chronic conditions, health care, that was once ‘waiting’, is now considered ‘initiative’ (Sun et al. 2023). As a result, nurses will increasingly find themselves treating patients in their own home, which could further amplify the phenomenon of violence, in a setting where nurses are extremely isolated and vulnerable (Campbell et al. 2013).

In order to design effective health policies and initiatives to mitigate the occurrence of the emerging issue of WPV, it is crucial to explore the prevalence of home care violence and its underlying determinants.

## 2 Background

WPV is one of the most impacting global issues health systems are currently experiencing, with patients and their caregivers (e.g., relatives) being the primary perpetrators of violence against nurses (Chakraborty et al. 2022). The WPV encompasses various forms of abuse, including physical, verbal, or psychological threats, mistreatment, coercion, or violence directed at nurses with the intent to endanger their safety, well‐being, or health, while they are performing their duties (Rossi et al. 2023).

Recent estimates identify WPV as the second leading cause of workplace fatality among women and the third most common cause of occupational accident deaths (Dadfar and Lester 2021). The accurate estimation of WPV prevalence is challenging due to the lack of a substantial nationally or internationally representative sample and the methodological heterogeneity between studies (Hanson et al. 2015). However, the WPV prevalence is widespread, likely underestimated, and varies among different study locations, healthcare settings, countries, and related geographical areas (Liu et al. 2019; Phoo and Reid 2022). A recent study by Bagnasco et al. (2024) found that the effects of WPV vary widely in hospital and community settings, with an increasing prevalence considering different countries (28.8% in Canada, 32.4% in Italy, 34.3% in Turkey, 38.8% in the United States, 44% in Honk Kong, 52% in Brazil and 54% in Lebanon). However, regardless of the geographical area, the World Health Organisation (WHO) estimates that between 8% and 38% of nurses experience at least one WPV episode during their career (Kafle et al. 2022). Notably, when focused on verbal abuse only, this percentage rises to 87% (Campbell et al. 2013). Moreover, WPV is highly likely to be underreported by nurses because of being resigned to the notion that violence is a part of their work (Bauersfeld and Majers 2023). This under‐representation seems to be consistent with other findings, which report a higher WPV rate, i.e., up to 50%, among home care nurses (Fujimoto et al. 2017). Physical, mental and professional WPV‐related events (e.g., post‐traumatic stress disorder, sleep disorder, reduction in commitment, productivity and job satisfaction, increased professional intention to leave) (Kafle et al. 2022; Kim et al. 2021; Pagnucci et al. 2022) can be detrimental to healthcare organisations, nursing staff as well as the standard and safety of patient care (de Raeve et al. 2023). WPV and its effects can also trigger an unending cycle of organisational inefficiencies and unsatisfactory patient experiences, which can set off further violent incidents (de Raeve et al. 2023).

The phenomenon of WPV has been widely studied in several healthcare settings, resulting in the interaction of complex factors that include patient, caregiver and healthcare staff, as well as the organisational environment (Pagnucci et al. 2022; Phoo and Reid 2022). Specific characteristics of the organisational environment seem to be associated with the aggression experienced by the staff during their shifts. These include workloads, number of patient accesses and the amount of overtime hours (Huang et al. 2020). There is evidence that nurses who work longer hours experience increased levels of work‐related stress, which in turn affects their technical performance (Yosiana et al. 2020) potentially contributing to violence incidents. Furthermore, demanding work environments, namely characterised by high workload levels, increase stress levels among nurses, leading to burnout (Dall'Ora et al. 2020) and decreased job satisfaction (White et al. 2020). Other variables have been associated with a higher incidence of violence against nurses, including sociodemographic and work‐related characteristics (e.g., gender, age, work experience, understaffing, high workload, and burnout) (Pagnucci et al. 2022). However, there is also evidence that the occurrence of such episodes can be reduced as a result of positive leadership, which can mitigate stress levels and enhance job satisfaction in nurses (Diehl et al. 2021; Senek et al. 2020). A rigid hierarchical structure, mixed with a low nurse manager ability, an increase in workload and the understaffing, can also lead to WPV (Pagnucci et al. 2022). Additionally, in community care settings where nurses provide services in patients' homes, factors such as multidisciplinary teams, while desirable for achieving a diverse skill mix, can also result in inconsistent communication and fragmented care plans, potentially eroding the trust that patients and their relatives have in the care team. This erosion of trust could escalate to episodes of violence (Bianconi et al. 2020).

Considering these fundamental premises, it is important to remember that nowadays healthcare is not limited to hospital environments. There is a growing trend in the shift of delivery of care from traditional care settings, such as hospitals, to noninstitutional care settings, such as the territory (Campbell et al. 2013). The factors that contribute to this global transition include population aging, the prevalence of chronic illnesses and disabilities, and the rising average life expectancy, which can result in higher costs and less effectiveness when patients are treated in hospitals (Fatemi et al. 2018; Hanson et al. 2015). As a result of this shift, there is a higher likelihood that nurses will do their care in patients' private homes, which poses a risk to their safety and well‐being, considering the dynamics and potentially dangerous factors that exist in this unpredictable setting of care (Nix and Altom 2023; Zhong and Shorey 2023). Care providers who are engaged in home care are isolated and more susceptible because they are not covered by the standard hospital safeguards and precautions (Geiger‐Brown et al. 2007). In confirmation of this, the statistics reveal that home health care providers, such as nurses, have some of the highest incidences of WPV compared to other professions (Campbell et al. 2013), both physical and verbal (Franz et al. 2010). According to a recent systematic review, over 30% of nurses had experienced at least one episode of WPV during their home care career (Phoo and Reid 2022).

Despite the general interest in WPV, there is evidence that this topic has comparatively fewer studies conducted in the context of community home care compared to those investigating hospital settings. Given the detrimental impact of WPV on public health systems and considering the actual general transition of nursing care from hospital to territory, it is crucial to investigate the prevalence and factors contributing to WPV in contexts of home care settings. This would help design more effective policies and execute more preventative and mitigating measures against these events (Kumari et al. 2022), especially in countries such as Italy, where this phenomenon is still unexplored. To our knowledge, only one systematic review was conducted to describe the international prevalence of WPV by identifying the determinants of violence towards care workers in the home setting (Phoo and Reid 2022). Furthermore, in the Italian context a scoping review was conducted to identify predictors of WPV and nursing students in mixed work environments (Pagnucci et al. 2022), and a recent cross‐sectional study sought to identify the protective and risk factors of WPV taking into consideration several settings of care (Bagnasco et al. 2024). However, to date, no primary research has ever been conducted in Italy to determine the prevalence of WPV or identify its determinants in the context of exclusive home care.

## 3 The Study

### 3.1 Aim(s)

The aim of the study is to measure prevalence and identify determinants of WPV in the Italian home care settings.

## 4 Methods

### 4.1 Design

This is a secondary analysis of data coming from the AIDOMUS‐IT study (acronym for ‘Assistenza Infermieristica Domiciliare in Italia’) (Bagnasco et al. 2022), a descriptive observational study whose aim was to describe the characteristics of the home care services in Italy. Briefly, the AIDOMUS‐IT study was a national cross‐sectional descriptive observational study conducted using a survey methodology. It incorporated three data sources: nursing directors, nurses and patients. Nurses from all participating community care centres were recruited through convenience sampling, with nursing coordinators from each centre serving as facilitators for the study. To map the characteristics of home care and identify existing gaps, data were collected on various aspects, including staffing, skill mix, work environment, workload, missed care, perceived psychosocial conditions in the workplace, WPV, job satisfaction, sociodemographic characteristics, and organisational factors. Specific organisational characteristics assessed included time dedicated to each home visit, kilometres travelled, and overtime work.

The reporting of this study followed the checklist reported in the STROBE guidelines (von Elm et al. 2014).

### 4.2 Study Setting and Sampling

The AIDOMUS‐IT study encompassed 70 Local Health Authorities situated in 18 Italian regions. This secondary analysis used data collected from the nurses directly involved in the home care services. More details of the AIDOMUS‐IT study can be consulted elsewhere (Bagnasco et al. 2022).

### 4.3 Inclusion and/or Exclusion Criteria

In order to be eligible for participation in the AIDOMUS‐IT study, nurses were required to have direct involvement in the provision of home care services at the time of data collection. Nurses who did not directly provide home care, such as those holding managerial or administrative positions, were not included in the study.

### 4.4 Conceptual Framework

**FIGURE 1:**
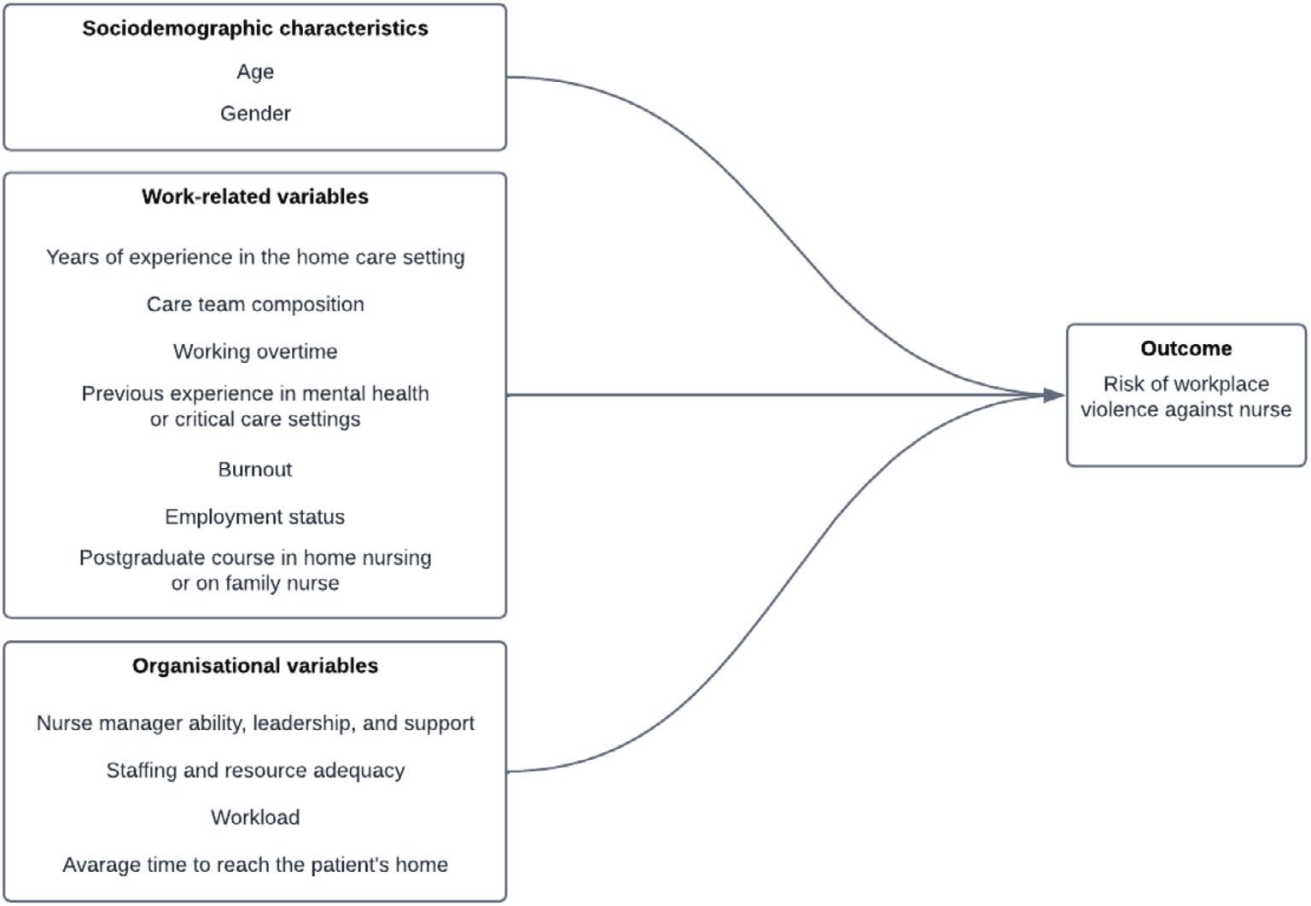
Conceptual framework of the study. [Colour figure can be viewed at wileyonlinelibrary.com]

Prior to data collection, we developed a conceptual framework to summarise the potential determinants of WPV in the home care setting. WPV have been defined as abuses put in place by individuals external to the organisation, specifically patients and/or their caregivers (Agenzia Europea per la Sicurezza e la Salute sul Lavoro 2018). Our approach involved three steps: (i) reviewing both theoretical and empirical research to identify the potential determinants; (ii) discussing any inconsistencies in findings with the research team; than (iii) reaching a final consensus to develop the model. The final conceptual framework is graphically displayed in Figure 1.

### 4.5 Instruments and Data Source

For the AIDOMUS‐IT study, a variety of instruments were used (Bagnasco et al. 2022). However, for the purposes of this secondary analysis only the following were employed, based on the conceptual framework developed.

#### 4.5.1 General Questionnaire

This tool was developed by the research team to collect information on the prevalence and type of violence episodes (i.e., verbal abuse or physical abuse) suffered in the 12 months prior to data collection. The questionnaire also contained specific sections to collect the sociodemographic and professional characteristics of the nurses (i.e., age, gender, educational attainment, years of experience in home care, critical care or mental health settings, presence of a postgraduate course attainment in home care nursing or family nurse, type of employment), as well as staffing characteristics (i.e., care team composition, frequency of overtime hours, and average time to reach the patient's home).

#### 4.5.2 Workload

Workload was measured with an adapted version of the National Aeronautics and Space Administration Task Load Index (NASA‐TLX) questionnaire. This is a 6‐item single‐dimensional tool in its original form, developed to investigate the subjective perception of nursing workload (Hart and Staveland 1988). Questions are formulated on a 20‐point response scale to investigate various aspects of performance across the dimensions of mental demand, physical demand, time demand, performance, effort, and frustration. In the AIDOMUS‐IT study, an adapted version of this tool, the NASA‐TLX_HCN‐IT was used (Zaghini et al. 2024). The instrument was assessed for validity and internal consistency, and includes six single‐dimensional items: mental demand, physical demand, temporal demand, emotional demand, performance, and frustration. The score was calculated by standardising from 0 to 100 the score obtained by summing all the items except the one referring to performance, where higher scores indicate a higher subjective workload.

#### 4.5.3 Burnout

Burnout was assessed with two questions from the COPSOQ‐III questionnaire. The COPSOQ‐III is a 29‐item tool to assess the perceived psychosocial conditions in the workplace (Burr et al. 2019), and it has been validated in Italian in a sample of health professionals (Peter et al. 2022). Questions are formulated on a 5‐point response scale ranging from 0 = never, to 4 = always. For this secondary analysis, we used the score reflecting the burnout dimension of the health and well‐being domain. Specifically, two questions were used: ‘in the last 4 weeks, how often have you been physically exhausted?’, and ‘in the last 4 weeks, how often have you been emotionally exhausted?’. These questions were chosen as they reflect important home care work environment factors. The total score ranges between 0 to 100, with higher scores reflecting higher burnout levels. The internal consistency of these two items was adequate in our data with a Cronbach's *α* value of 0.81.

#### 4.5.4 Staff Adequacy and Nurse Manager Ability, Leadership, and Support

Staff adequacy and nurse manager ability, leadership, and support were measured with the PES‐NWI. This is a 32‐item tool to assess the perceived quality of the work environment (Lake 2002; Zanini et al. 2022). Questions are formulated on a 5‐point Likert scale ranging from 1 = strongly disagree, to 4 = strongly agree. In this study, we used the scores related to the specific dimensions of perceived staff and resource adequacy, and leadership capacity of the nurse manager (total of nine questions), where values above 2.5 indicate a more favourable environment in the corresponding dimension. In our study, both the dimensions of perceived staff adequacy and leadership capacity of the nurse manager showed strong internal consistency, with Cronbach's *α* values of 0.88 and 0.91, respectively.

### 4.6 Data Collection

After receiving the approval of the study by the primary Institutional Review Board, the nurses were notified by their respective managers from each Health Local Authority about the opportunity to participate in the study, and that participation was voluntary. An online informative report (via the LimeSurvey Web application) was made available to the potentially eligible nurses, who were also required to sign an informed consent form before the beginning of the survey.

### 4.7 Data Analysis

Descriptive statistics (i.e., means, standard deviations, frequencies, and percentages) were used to describe the general characteristics of the sample. To investigate the determinants associated with WPV, a multivariable binary logistic regression model was performed. Independent variables were selected considering existing literature and through discussions within the research team. A post hoc power analysis was conducted to assess whether the sample size was sufficient for reliable statistical inference. The required sample size was estimated following a formula for logistic regression, which includes a minimum sample size of 100 + 50i, where ‘i’ represents the number of independent variables included in the model (Bujang et al. 2018). Given that our study incorporated 13 independent variables, this formula indicated that a minimum of 750 nurses was necessary to ensure adequate statistical power.

Dummy variables were used where necessary (e.g., for team composition) to avoid model over‐fitting. All the independent variables were checked for possible multicollinearity (using variance‐inflation factor—VIF) before entering the regression model. Effect sizes were reported as adjusted odds ratios (aOR) along with 95% confidence intervals (CIs) and their *p*‐values. All the analyses were conducted using R‐studio v.4.2.3 (R Core Team 2013).

### 4.8 Ethical Considerations

The AIDOMUS‐IT study was approved by the primary Institutional Review Board on 11/29/2022, under the protocol number 675/2022—dB ID 12844. Data confidentiality has been strictly maintained. Informed consent was obtained from all participants.

## 5 Results

### 5.1 Characteristics of the Sample

A total of 3949 nurses participated in this study. The sample had a mean age of 46.02 years (SD = 10.23) was mostly female (78.20%), with an average home care setting experience of 8.01 years (SD = 8.26). The perceived workload was moderate (M = 47.85, SD = 21.69), as well as burnout levels (M = 51.78, SD = 24.03). Overall, the number of human and material resources in the workplace was perceived as appropriate, since 61.56% of the sample reported a PES‐NWI staffing adequacy score > 2.5. According to 83.39% the respondents, nurse managers' leadership style and ability to support nursing staff was perceived positive by the nurses (PES‐NWI nurse manager ability score > 2.5). Regarding the organisational factors, most respondents reported being a public employee (93.49%) and working in a multiprofessional team composed by nurses and other healthcare providers (62.02%). The average minutes required by nurses to reach the patients' homes was 34.70 (SD = 37.16), and almost one‐third of participants reported working overtime (36.72%). Overall, 809 nurses (20.49%) reported to have experienced an episode of violence in the last 12 months; of this, 96.93% of the sample reported verbal abuse, while 14.39% reported physical abuse. The characteristics of participants are summarised in Table 1, while Table 2 reports details regarding WPV occurred.

**TABLE 1:** Characteristics of the participants.

Characteristics	Total (*n* = 3949)	WPV in the past 12 months (*n* = 809)	No WPV in the past 12 months (*n* = 3140)
Age (years), mean (SD)	46.02 (10.23)	44.84 (10.37)	46.33 (10.17)
Gender, *n* (%)			
Male	784 (19.85)	144 (17.80)	640 (20.38)
Female	3088 (78.20)	649 (80.22)	2439 (77.68)
Prefer not to say	77 (1.95)	16 (1.98)	61 (1.94)
Years of experience in the home care setting (years), mean (SD)	8.01 (8.26)	7.34 (7.63)	8.19 (8.41)
Care team composition, *n* (%)			
Nurses only	1500 (37.98)	287 (35.48)	1213 (38.63)
Nurses and other healthcare providers	2449 (62.02)	522 (64.52)	1927 (61.37)
Working overtime (yes), *n* (%)	1450 (36.72)	339 (41.90)	1111 (35.38)
Previous experience in mental health or critical care settings, *n* (%)	1303 (33.00)	281 (34.73)	1022 (32.55)
Burnout score^a^, mean (SD)	51.78 (24.03)	58.59 (22.93)	50.02 (24.00)
Employment status, *n* (%)			
Public employment	3692 (93.49)	754 (93.20)	2938 (93.57)
Professional cooperative	121 (3.06)	22 (2.72)	99 (3.15)
Freelance	136 (3.44)	33 (4.08)	103 (3.28)
Postgraduate course in home nursing or on family nurse, *n* (%)	1023 (25.91)	231 (28.55)	792 (25.22)
PES‐NWI			
Nurse manager ability, leadership and support (score < 2.5), *n* (%)	656 (16.61)	183 (22.62)	473 (15.06)
Staffing and resource adequacy (score < 2.5), *n* (%)	1518 (38.44)	373 (46.11)	1145 (36.46)
NASA‐TLX, mean (SD)	47.85 (21.69)	50.96 (21.73)	47.04 (21.16)
Minutes to reach the patients' homes, mean (SD)	34.70 (37.16)	35.15 (37.22)	34.58 (37.14)

Abbreviations: NASA‐TLX, NASA Task Load Index; PES‐NWI, Practice Environment Scale of the Nursing Work Index; SD, standard deviation; WPV, workplace violence against nurses.

^a^ Measured with the Copenhagen Psychosocial Questionnaire III.

**TABLE 2:** Descriptives of the episodes of violence (*n* = 809).

	*n* (%)
Number of verbal abuses suffered	
None	22 (3.07)
One	305 (42.54)
Two	191 (26.64)
Three or more	199 (27.75)
Number of physical abuses suffered	
None	613 (85.61)
One	68 (9.50)
Two	23 (3.21)
Three or more	12 (1.68)
Reported the episode of violence suffered	534 (73.76)

### 5.2 Determinants of Workplace Violence

The results of the logistic regression model revealed that some factors were associated with an increased risk of violence in the nursing workplace. Regarding sociodemographic characteristics of nurses, age was found to be a significant factor associated with episodes of violence. Specifically, for each additional year of age, the odds of experiencing an episode of violence decreased by 2% (aOR = 0.98, *p* = 0.002). Several organisational factors were associated with an increased risk of violence in the nursing workplace. For each one‐unit increase in workload, the odds of experiencing an episode of violence increased by 1% (aOR = 1.01, *p* = 0.002). Nurses working in a multiprofessional team (nurse assistants, physicians and other healthcare professionals), compared to nurses working in a team consisting of only nurses, were 24% more likely to experience an episode of violence (aOR = 1.24, *p* = 0.018), while nurses that reported an inadequate nurse manager ability, leadership, and support were 38% more likely to experience an episode of violence (aOR = 1.38, *p* = 0.003). Finally, for each one‐unit increase in burnout score, the odds of experiencing an episode of violence increased by 1% (aOR = 1.01, *p* < 0.001) (Table 3).

**TABLE 3:** Multivariable binary logistic regression analysis for factors associated with an increasing risk of violence in the nursing workplace (*n* = 3949).

	aOR	95% CI	*p*	VIF
Age (years, 1 unit increase)	0.98	0.98–0.99	**0.002**	1.36
Gender (male vs. female)	0.87	0.70–1.09	0.225	1.05
Gender (prefer not to answer vs. female)	0.86	0.44–1.58	0.648	1.05
Years of experience in the home care setting (years, 1 unit increase)	0.99	0.98–1.01	0.276	1.29
Care team composition (nurses and other members vs. nurses)	1.24	1.04–1.48	**0.018**	1.02
Working overtime (yes vs. no)	1.08	0.90–1.30	0.371	1.08
Previous experience in mental health or critical care settings (yes vs. no)	1.16	0.97–1.39	0.101	1.04
Burnout (total score, 1 unit increase)^a^	1.01	1.01–1.02	**< 0.001**	1.17
Employment status (professional cooperative vs. public employment)	0.84	0.49–1.37	0.495	1.08
Employment status (freelance vs. public employment)	1.14	0.69–1.80	0.600	1.08
Postgraduate course in home nursing or on family nurse (yes)	1.12	0.93–1.36	0.238	1.03
PES‐NWI Nurse Manager ability, leadership and support score (< 2.5 vs. > 2.5)	1.38	1.11–1.70	**0.003**	1.05
PES‐NWI Staffing and resource adequacy score (< 2.5 vs. > 2.5)	1.09	0.90–1.31	0.357	1.16
NASA‐TLX (total score, 1 unit increase)^b^	1.01	1.00–1.01	**0.002**	1.06
Minutes to reach the patients' homes (1 unit increase)	1.00	0.99–1.00	0.648	1.02

*Note:* The dependent variable was dummy coded as follows: 0 = nurses not reporting episodes of violence in the previous 12 months, 1 = nurses reporting at least one episode of violence in the previous 12 months. Significant *p*‐values are in bold. Null deviance: 3491.2; Residual deviance: 3368.3 on 3422° of freedom; AIC: 3400.3; Tjur's *R* ^2^: 0.04.

Abbreviations: aOR, adjusted odds ratio; *p*, *p*‐value; SE, standard error; VIF, variance‐inflation factors.

^a^ Measured with items from the Copenhagen Psychosocial Questionnaire III. Higher scores indicate higher burnout.

^b^ Higher scores indicate a higher workload.

## 6 Discussion

The purpose of this study was to report the prevalence and determinants of WPV in the Italian home care settings. We found that approximately one in five nurses reported experiencing WPV from either patients or their caregivers within the 12 months before data collection. However, drawing comparisons with the existing literature poses significant challenges due to the heterogeneous data and potential underreporting issues regarding WPV prevalence (Liu et al. 2019). Despite these obstacles, the estimates found in the literature seem to align with the data obtained in the AIDOMUS‐IT study regarding home care nurses (Liu et al. 2019). This represents a notable concern, especially considering the global transition from hospital‐centred to home‐based care settings, where standard safety measures may be lacking. Moreover, WPV carries several concerning consequences on nurses, healthcare systems and, ultimately, on patients (Mento et al. 2020).

The shift towards home‐based care, which is already underway in Italy (Ministero della Salute 2022), prompted a secondary analysis of the AIDOMUS‐IT data. This analysis sought to investigate the influence of nurses' characteristics, work‐related aspects, and organisational features on the occurrence of WPV. While existing literature suggests that predictors of violence towards home care workers encompass client, worker, and organisational features (Pagnucci et al. 2022; Phoo and Reid 2022), patients' characteristics were not assessed in this analysis. In fact, the AIDOMUS‐IT study primarily focused on evaluating the strengths and weaknesses of Italian home care services. Consequently, this analysis focused on exploring the impact of attributes of nurses and services on the occurrence of WPV, with the aim of outlining areas for improvement. Moreover, patients' data were not considered starting from the assumption that provided home care services were flexible and customised according to patients' characteristics and needs, minimising the occurrence of WPV related to patients' unmet needs. Nevertheless, further studies may consider also patient's data, evaluating additional elements such as the patient's health literacy level, which has been demonstrated over time to be related to the complexity of care (Cocchieri et al. 2024) and, when low, to the incidence of WPV (Chakraborty et al. 2022).

The profile emerging from the regression analysis of nurses more prone to undergo WPV included individual, work, and organisational features. These determinant, listed from strongest to weakest, were perceived inadequate nurses' manager abilities of leadership and support, presence of professionals other than nurses in the team, lower age, higher perceived workload, and higher burnout levels among nurses. The literature agrees that a strong leadership fostering inclusivity, support, and respect is fundamental to prevent WPV (Fricke et al. 2023). This effect is probably mediated by the ability of the leader to listen to their collaborators' needs, as also suggested by the contents of the PES‐NWI employed in this study (Lake 2002), to evaluate nurses' perceived work environment. These reflections, along with literature results (Swiger et al. 2017), suggest that specific assessments and actions should be undertaken within Italian healthcare facilities. In particular, it is suggested both the assessment of leadership styles adopted in home care services, and the development of strategies to improve leadership, other than effective programs for the prevention and prompt treatment of WPV, including educational courses and rapid support for involved professionals (Civilotti et al. 2021; Liu et al. 2019). The development of appropriate leadership is even more worthwhile when considering the high amount of available literature documenting its influence also on other nurses', patients', and organisational outcomes (Swiger et al. 2017).

Difficulties in building a solid teamworking strategy among different healthcare professionals may have led to care inconsistency (Babiker et al. 2014; King et al. 2008) and, hence, lesser ability to meet patients' needs who, in turn, may have developed aggressive behaviours. In this regard, it is important to note that building a team requires specific strategies and commitment (King et al. 2008), and that patients' trust in healthcare professionals seems to have an association with better patient‐reported health outcomes, such as care satisfaction, health‐oriented behaviours, and quality of life (Birkhäuer et al. 2017). Moreover, it should be considered that patients cared from a multidisciplinary team probably had more intensive and complex needs, leading to higher probability to experience frustration during their care pathway. This may explain the determinant role of the multidisciplinary team in the occurrence of violence. In addition, the access of different and several professionals at patients' home may have been experienced by patients as a practice too invasive of their privacy. Hence, it may be useful to improve team‐building and effective handover strategies among different professionals involved in home care (Desmedt et al. 2021), other than developing and adhering to specific standards for home care, especially in care planning and organisation (Sabetsarvestani et al. 2022).

Surprisingly, gender did not show a determinant role in WPV occurrence, as opposed to age, showing early‐career nurses to be more at risk of violence. It could be hypothesised, also considering literature data, that the role of this determinant was connected to nurses' longer experience in home care, or previous experience in emergency or mental health settings, or having had specific education for home care, or even to the type of contract, i.e., freelance nurses could be less engaged to the organisation (Liu et al. 2019; Pagnucci et al. 2022; Phoo and Reid 2022). However, this was not the case, since all the above‐mentioned variables did not show a determinant role towards the outcome, suggesting that advanced‐career nurses simply acquired softer skills due to longer management of human beings' needs and age‐related wisdom. Hence, it is hard to hypothesise interventions that could modulate the role of this determinant; however, involving advanced‐career nurses in coaching early‐career colleagues regarding their attitudes at patients' home may be an option.

Finally, workload and nurses' burnout probably affected the ability to establish a close relationship with patients, including empathy and an adequate consideration of patients' needs (Phoo and Reid 2022), resulting in patients' perception to be neglected. However, working overtime, nurses' perception of staff adequacy, and time to reach patients' house did not emerge as determinants. Hence, it seems that when nurses perceived to provide adequate care at patients' homes and effectively met their needs, several other aspects felt a part and did not have a significant effect in the establishment of a good climate able to mitigate violence occurrence. In this regard, it is advisable to estimate optimum staffing to avoid inadequate workload, and monitor nurses' burnout (Dall'Ora et al. 2020), along with support interventions for colleagues experiencing such syndrome.

AIDOMUS‐IT is the first national study, involving a numerous sample, and investigating features, weaknesses, and strengths of home care in Italy, and this is its main strengths, which could be extended also to this secondary analysis. However, this study has some limitations. The cross‐sectional design, which allowed to measure exposure and outcome at the same time, is usually considered to be only marginally relevant for drawing conclusions about causality. As a matter of fact, cross‐sectional studies are typified by their incapacity to distinguish between a putative cause and its potential effect; a substantial correlation between two concepts does not imply that one causes the other (Savitz and Wellenius 2023). Consequently, it is important to interpret the findings of our study carefully. In addition, a bias‐response may have been produced by using self‐reported tools and data (Rosenman et al. 2011). In addition, a main limitation of this analysis is the unavailability of data on patients or caregivers perpetrating violence (e.g., health literacy levels). Moreover, other variables related to nurses (education level, other psychological aspects not included here) that could impact on WPV were not included in the model, thus they may have completed the figure regarding determinants of WPV. Hence, more research is needed to develop an exhaustive framework regarding antecedents of this phenomenon, other than to assess the effectiveness of the proposed corrective measures.

## 7 Conclusion

WPV against nurses has shown a concerning prevalence among Italian home care nurses, with implications for the nurses themselves, the healthcare system, and the patients. These findings call for immediate action to address the phenomenon by mitigating its determinants and further investigating its antecedents.

Insights from this secondary analysis conducted on AIDOMUS‐IT data reveal that many determinants of WPV are modifiable or monitorable. Specifically, it is recommended to establish effective leadership that fosters inclusivity and actively listens to nurses' viewpoints. Moreover, it is also advisable to support colleagues who have experienced the phenomenon, addressing predisposing syndromes such as burnout, and involving advanced‐career nurses in developing the soft skills of early‐career colleagues. Mentorship can create a supportive environment and enhance preparedness for potential WPV incidents. Adequate nurses' workload and effective team‐building strategies should also be prioritised to prevent conditions that could exacerbate WPV, potentially through the intermediate role of unmet patients' needs.

Further research within the Italian context is necessary to investigate the role of patients' characteristics in the occurrence of this phenomenon, other than to complete the reference framework for WPV occurrence, and to assess the outcomes of proposed coping strategies.

## Author Contributions

The final draft has been approved by all authors, who also satisfy the following requirements. Francesco Zaghini, Manuele Cesare, Marco Di Nitto, Paolo Iovino, Valeria Caponnetto: Made substantial contributions to the conception and design and were involved in drafting the manuscript. Marco Di Nitto: Made substantial contributions to the acquisition, analysis, or interpretation of data. Alessandra Burgio, Loredana Sasso: Involved in revising the manuscript critically for important intellectual content. Annamaria Bagnasco, Alessandra Burgio, Beatrice Mazzoleni, Duilio Fiorenzo Manara, Francesco Zaghini, Giancarlo Cicolini, Gennaro Rocco, Ilaria Marcomini, Loreto Lancia, Laura Rasero, Loredana Sasso, Manuele Cesare, Marco Di Nitto, Maurizio Zega, Paolo Iovino, Paolo Landa, Rosaria Alvaro, Valeria Caponnetto, Yari Longobucco: Given final approval of the version to be published.

## Disclosure

The authors have checked to make sure that our submission conforms as applicable to the Journal's statistical guidelines. The statistics were checked prior to submission by an expert statistician (Marco Di Nitto). The author(s) affirm that the methods used in the data analyses are suitably applied to their data within their study design and context, and the statistical findings have been implemented and interpreted correctly. The author(s) agrees to take responsibility for ensuring that the choice of statistical approach is appropriate and is conducted and interpreted correctly as a condition to submit to the Journal.

## Ethics Statement

This study protocol was approved by the Liguria Ethics Committee (Ref. N. 675/2022—dB id 12844).

## Conflicts of Interest

The authors declare no conflicts of interest.

## Supporting information


Data S1.

## Data Availability

Due to ethical and privacy restrictions, data are only available upon reasonable request.
